# Case Report: A rare case of giant ascending aortic dissecting aneurysm requiring redo sternotomy for rescue

**DOI:** 10.3389/fcvm.2025.1656155

**Published:** 2025-10-22

**Authors:** Biao Wang, Qi Wang, Zengshan Ma, Xin Zhao

**Affiliations:** Department of Cardiovascular Surgery, Qilu Hospital, Cheeloo College of Medicine, Shandong University, Jinan, Shandong, China

**Keywords:** aortic dissection aneurysm, type a aortic dissection, re-openoperation, complication, Bentall operation

## Abstract

**Background:**

Here, we report a rare case of a 45-year-old female with a giant ascending aortic dissecting aneurysm (diameter >10 cm) who underwent a redo sternotomy for rescue.

**Methods:**

The patient had aortic coarctation repair and mechanical aortic valve replacement 20 years earlier. Although the patient had significant surgical risks due to a low hemoglobin level and long-term warfarin use, urgent surgical intervention was deemed necessary. The surgery involved cannulating the femoral artery and vein, splitting the sternum, and carefully dissecting the aneurysm. A 23-mm valve conduit was implanted, and the left coronary ostium was transplanted. The right coronary ostium was anastomosed using the Cabrol technique.

**Results:**

The surgery was successful, with a total operative time of 4 h and 25 min, cardiopulmonary bypass time of 143 min, and aortic cross-clamp time of 78 min. Postoperatively, the patient experienced frequent episodes of cardiac arrest, which resolved with the implantation of a temporary pacing device. Postoperative imaging showed satisfactory results, and the patient was discharged on the ninth postoperative day.

**Conclusion:**

This case reports the successful management of a complex and high-risk surgical scenario and provides valuable insight for similar cases.

## Introduction

Giant ascending aortic dissecting aneurysms with a diameter exceeding 10 cm are exceedingly rare (1–3), and very few cases of redo sternotomy surgery have been reported. This is the first report of the occurrence of such a large aneurysm following aortic coarctation and mechanical aortic valve replacement. The aortic dissection's false lumen showed seepage, but it did not lead to massive bleeding or death.

## Method

**Figure 1:**
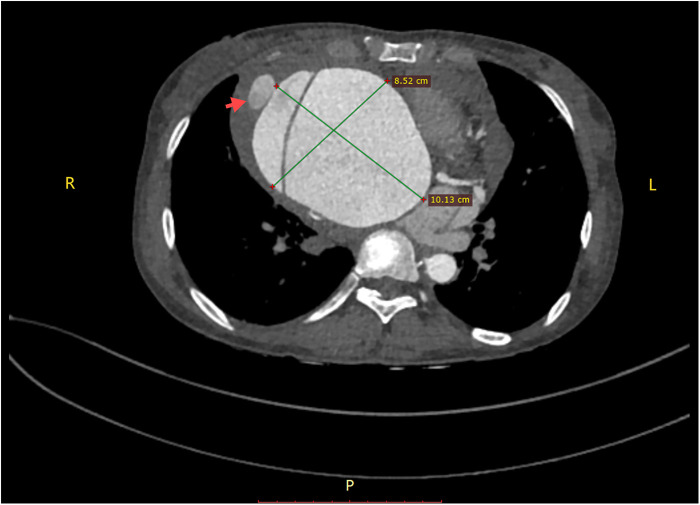
A giant aortic dissecting aneurysm. Contrast medium was observed to leak from the false lumen (red arrow).

**Figure 2:**
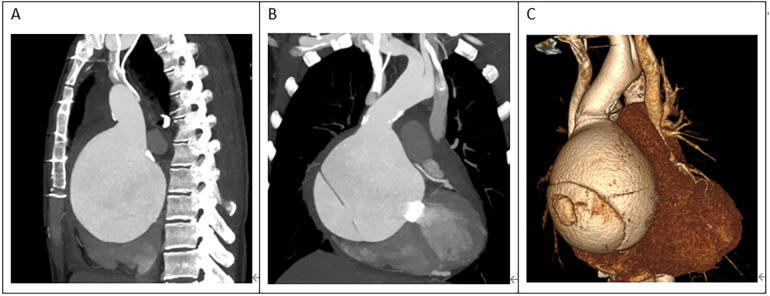
**(A)** The ascending aortic aneurysm was extremely large, occupying almost the entire mediastinum. **(B,C)** The heart was severely compressed.

A 45-year-old female patient, with a height of 155 cm and a low weight of 42 kg, was admitted to the hospital due to severe chest and back pain that had persisted for 9 h. The patient had undergone mechanical aortic valve replacement and aortic arch coarctation repair at our hospital 20 years earlier (the two operations were performed concomitantly). She had been taking warfarin for long time. She rarely received follow-up after surgery. Upon admission, the patient exhibited significant pain symptoms, accompanied by chest tightness, shortness of breath, and difficulty breathing; however, her vital signs were relatively stable, with a heart rate of 90–100 beats per minute. The physical examination revealed obvious surgical scars on the chest and left side of the thorax. Mechanical valve opening and closing sounds could be heard in the precordial area, along with a 3/6 grade systolic murmur. The electrocardiogram showed sinus rhythm, regular rhythm, and complete right bundle branch block. Echocardiography revealed a giant ascending aortic dissection aneurysm. The mean pressure gradient across the original mechanical aortic valve was 30 mmHg, with a peak gradient of 56 mmHg. The left ventricular diameter was 40 mm, the left atrial diameter was 19 mm, and the left ventricular ejection fraction (LVEF) was 70%. The enhanced computed tomography angiography (CTA) scan supported the presence of a giant ascending aortic dissecting aneurysm, with the largest cross-sectional area measuring approximately 101.3 × 85.2 mm (Figure 1). Contrast medium was observed to leak from the false lumen (Figure 1). The patient was slender, and the aneurysm occupied almost the entire anterior and posterior mediastinum (Figure 2A), severely compressing the heart (Figures 2B,C).

**Figure 3:**
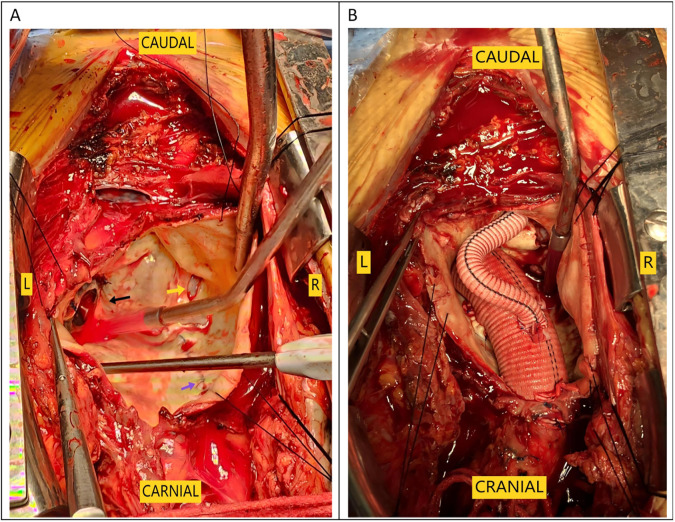
Intraoperative images. **(A)** The black arrow indicates the original prosthetic valve, the yellow arrow indicates the aortic dissection tear, and the purple arrow points to the aortic incision from the first surgery. **(B)** Cabrol anastomosis was performed at the right coronary ostium.

The surgical risk was significant, given the patient's severe condition and the large size of the aneurysm, as well as her low hemoglobin level of 68 g/L and long-term warfarin use. However, urgent surgical intervention was deemed necessary to save her life. Through departmental discussion, it was decided to establish extracorporeal circulation by cannulating the femoral artery and vein first. Assisted by extracorporeal circulation, the sternum was split, and the tissues behind the sternum and around the ascending aortic aneurysm were carefully dissected. The patient had a normal-diameter aorta in the upper segment of the ascending aorta, providing sufficient space for occlusion. The ascending aortic aneurysm was directly incised, which revealed an approximately 15-mm-sized rupture within the aneurysm (Figure 3A). The left coronary ostium was relatively large, with a diameter of about 8 mm, and cardioplegic solution was directly perfused. The right coronary ostium was very small, with a diameter of about 1 mm, and was considered to have essentially lost function. The original mechanical valve was removed, and a 23-mm valved conduit (Abbott Company, vessel diameter 25 mm) was implanted. The left coronary ostium was directly transplanted, while the right coronary ostium was anastomosed to an 8 mm artificial vessel using the Cabrol technique (Figure 3B). The artificial vessel was wrapped with autologous vascular wall tissue.

## Results

**Figure 4:**
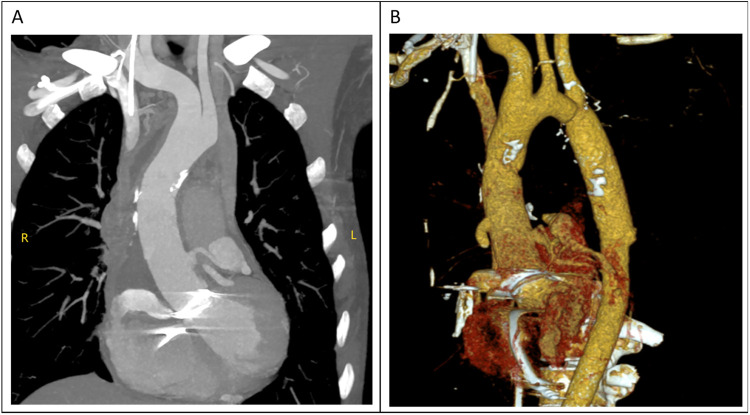
**(A,B)** the diameter of the ascending aorta had returned to normal in the computed tomography (CT) examination performed after surgery.

The total surgical time was 4 h and 25 min, with a cardiopulmonary bypass time of 143 min and an aortic cross-clamp time of 78 min. The tracheal tube was removed 13 h postoperatively. However, the patient began to experience frequent episodes of cardiac arrest lasting 5–10 s during the 20th and 25th hours postoperatively, for a total of 12 episodes, all of which reversed spontaneously. The cardiac conduction tissue was suspected to be damaged or affected by edema. An emergency temporary pacing device was implanted, and no further episodes of cardiac arrest occurred; her condition remained stable. Postoperative CTA scan showed satisfactory revision results (Figures 4A,B), and the postoperative echocardiogram demonstrated normal valve function. The patient was discharged on the ninth postoperative day.

## Discussion

**Figure 5:**
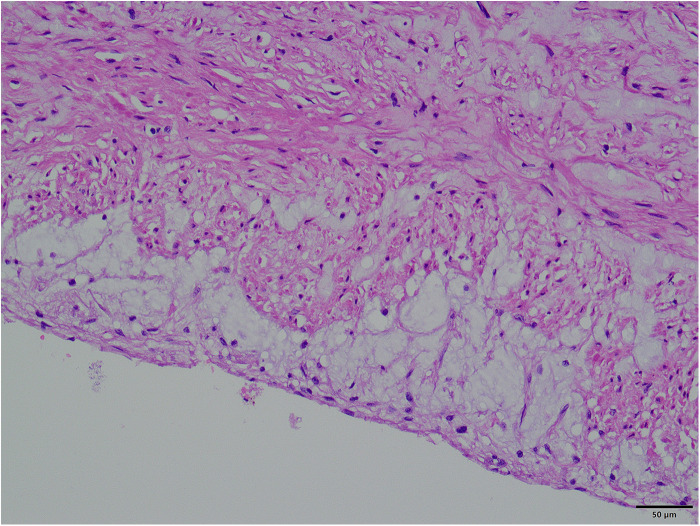
Pathological picture of the aneurysm wall (×400).

The surgical approach for this patient was the conventional method usually used by our team for second sternotomy surgery. However, some aspects are worth analyzing. One question is why the aneurysm grew so large in 20 years. Patients with some diseases, such as Marfan syndrome, vascular Ehlers-Danlos syndrome, Turner syndrome, and Loeys-Dietz syndrome, have a high risk of aneurysm expansion, dissection, and rupture (3, 4). The patient in this case had a congenital aortic developmental abnormality and had undergone surgical treatment. However, since the diameter of other blood vessels was normal (including at the site of the previous surgery), the ascending aortic tissue could have had a congenital developmental abnormality. Our pathology report also indicated that the ascending aortic wall had fibrous connective tissue hyperplasia and lacked normal elastic fiber tissue (Figure 5). During surgery, we found that the patient's previous surgical incision had shifted to the posterior wall of the aneurysm (Figure 3A), indicating that the enlargement of such a large aneurysm may be asymmetric, not coaxial, and is the first time this phenomenon was observed in such patients. The fact that such a large aneurysm grew in such a slender patient without rupture may be related to the patient's previous cardiac surgery, in which adhesions formed around the aorta, preventing acute rupture (5, 6).

Another important question is why the patient experienced frequent episodes of cardiac arrest after surgery. Similar cases appear in previous reports in which conduction problems were observed and required the implantation of a pacemaker (7). These reports indicate that conduction problems can occur in such patients, perhaps due to conduction damage. Aortic valve implantation surgery is easy to perform in patients with a large aortic sinus and a normal aortic annulus. However, an oversized artificial valve may affect the surrounding tissue, causing damage to the conduction system. Damage to the conduction system from aortic valve replacement surgery may be permanent, and can result in permanent third-degree atrioventricular blockage (which we have encountered); however, some damage is less severe, and conduction function may recover after treatment. The conduction function of the patient in this case was normal at discharge, and she has not experienced any further cardiac arrest episodes. However, regular, close follow-up is needed.

The patient's right coronary artery was underdeveloped. This could be due to the chronic enlargement of the ascending aortic aneurysm, leading to chronic occlusion of the right coronary artery. Postoperative coronary CTA examination indicated that the right coronary artery was very small. However, the left coronary artery was quite large. Therefore, no ischemic manifestations were observed in the heart, and the patient had no corresponding symptoms.

In summary, this report of the successful rescue of a complex and critical case involving a patient with a giant ascending aortic aneurysm dissection, provides valuable experience for treating similar patients in the future.

## Data Availability

The original contributions presented in the study are included in the article/Supplementary Material, further inquiries can be directed to the corresponding author.
