# Supplementary figures and images for: Case Report: A rare case of giant ascending aortic dissecting aneurysm requiring redo sternotomy for rescue
Source: Front Cardiovasc Med. 2025 Oct 22;12:1656155. doi: 10.3389/fcvm.2025.1656155 (PMC12585939; doi:10.3389/fcvm.2025.1656155)

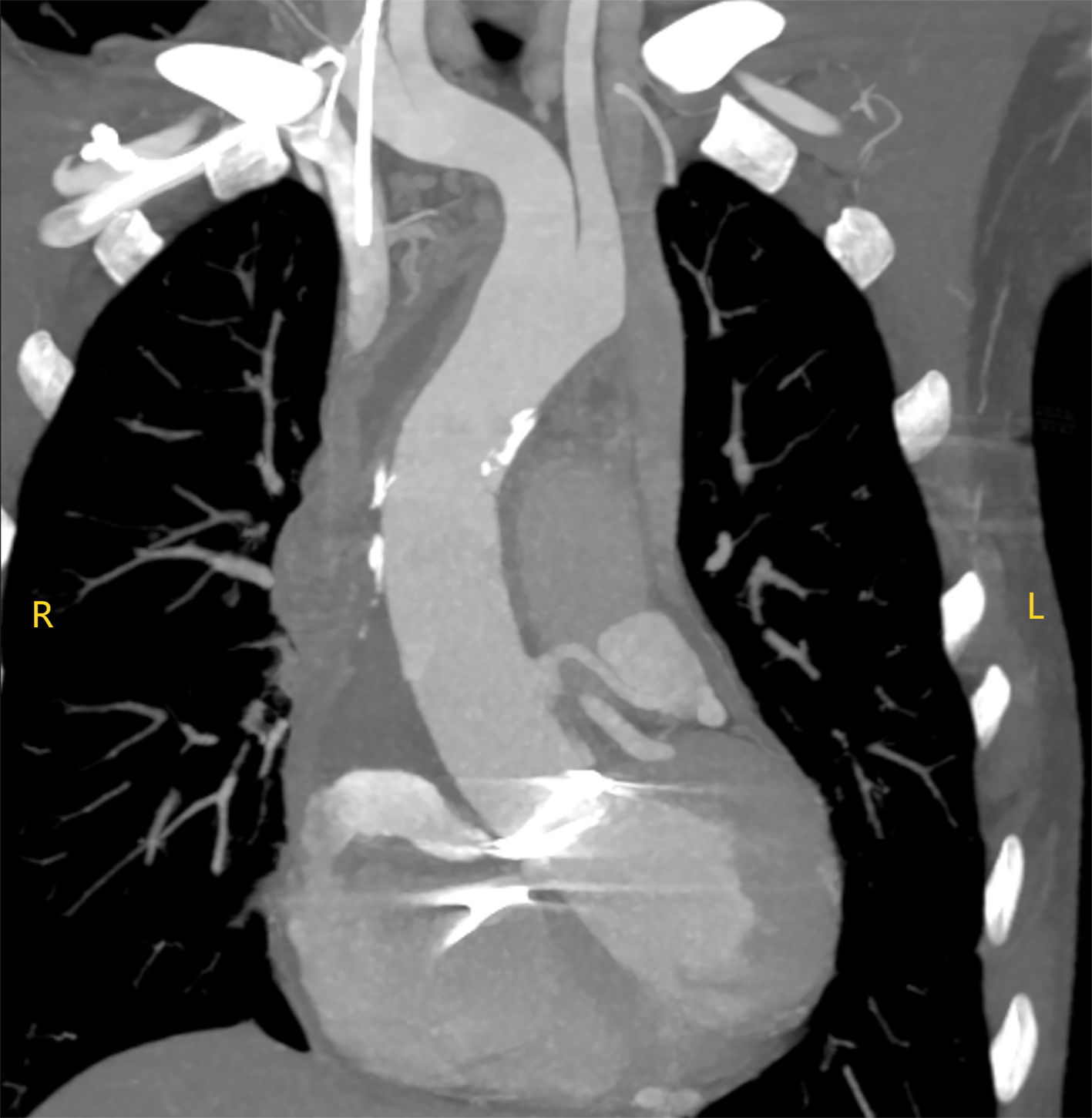

Supplement: Supplementary file 1 [file Image1.tif]

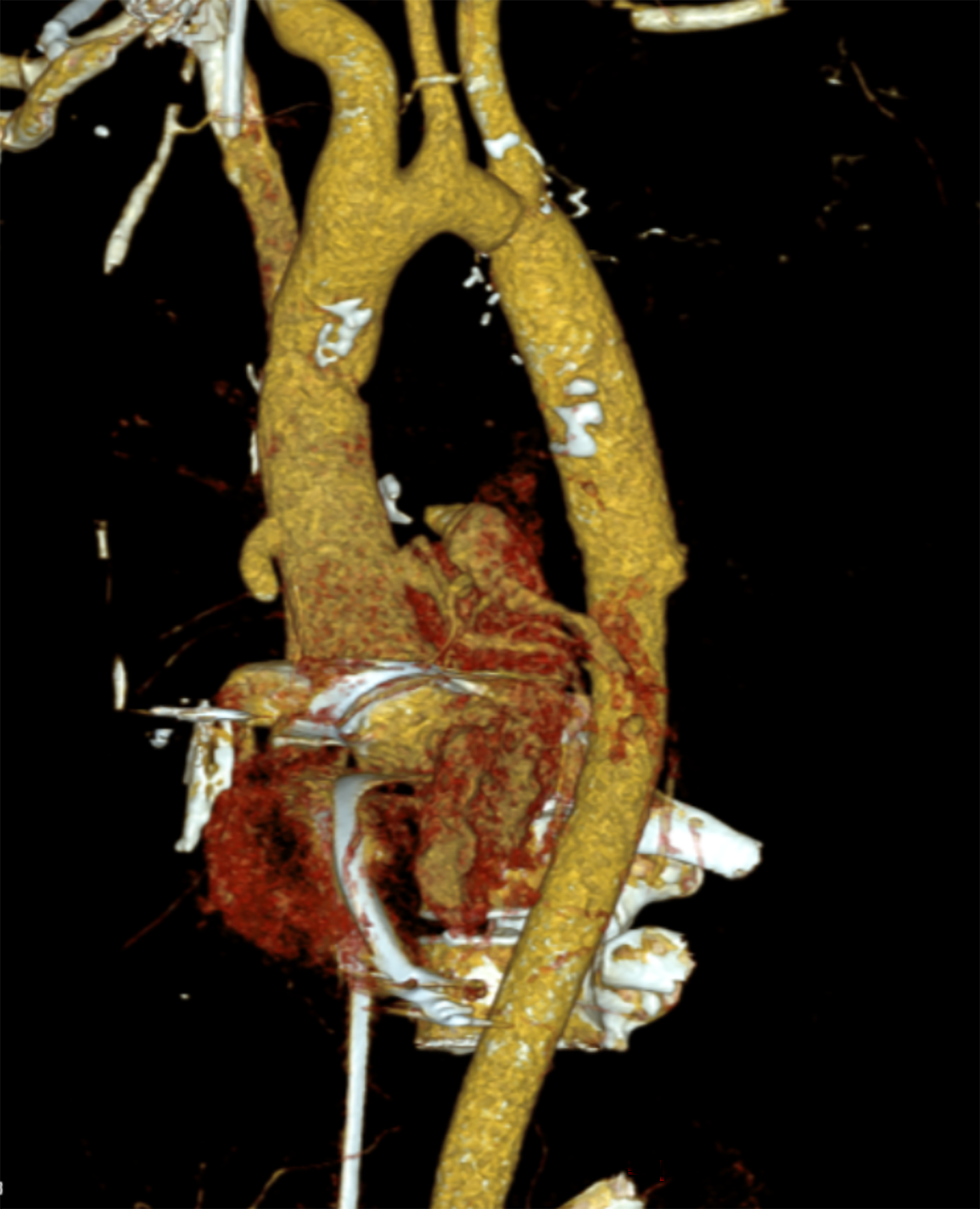

Supplement: Supplementary file 2 [file Image2.tif]

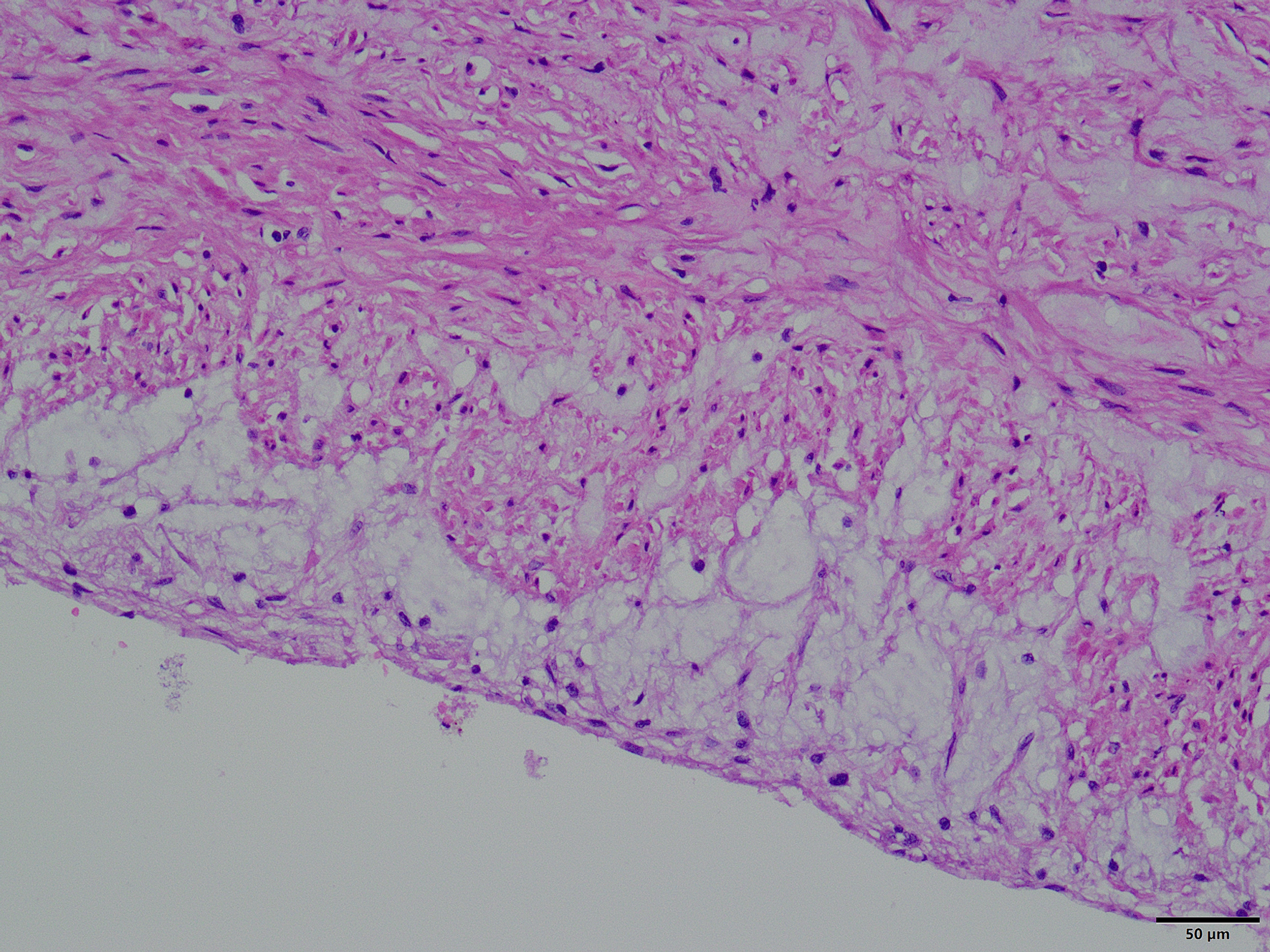

Supplement: Supplementary file 3 [file Image3.tif]
